# Evolutionary Changes of Hepatitis B Virus Pre-S Mutations Prior to Development of Hepatocellular Carcinoma

**DOI:** 10.1371/journal.pone.0139478

**Published:** 2015-09-30

**Authors:** An-Ye Zhang, Ching-Lung Lai, Fung-Yu Huang, Wai-Kay Seto, James Fung, Danny Ka-Ho Wong, Man-Fung Yuen

**Affiliations:** 1 Department of Medicine, The University of Hong Kong, Hong Kong SAR, China; 2 State Key Laboratory for Liver Research, The University of Hong Kong, Hong Kong SAR, China; Kaohsiung Medical University Hospital, Kaohsiung Medical University, TAIWAN

## Abstract

**Background and Aims:**

Deletions/mutations in the hepatitis B virus (HBV) pre-S region have been associated with hepatocellular carcinoma (HCC). We aimed to study the evolutionary changes of pre-S mutations prior to HCC development.

**Methods:**

We studied the HBV pre-S sequences at 1 to 10 years preceding diagnosis of HCC in 74 patients with HBV-related HCC (HCC group). 148 chronic hepatitis B patients matched for sex and age in 2:1 ratio, who had been followed up for at least 3 years without HCC (HCC-free group) were recruited as controls. 56 and 47 patients of HCC and HCC-free groups respectively had serially stored sera for longitudinally examination at 1–3 years, 4–6 years, 7–9 years and ≥10 years prior to the recruitment of the study.

**Results:**

Compared to the HCC-free group, higher frequencies of pre-S deletions and point mutations (at 11 codons) were observed in the HCC group (p<0.05). Multiple logistic regression analysis showed that pre-S deletions, point mutations at codon 51 and 167 were independent factors associated with HCC. Longitudinal observation showed that pre-S deletions and most of the 11 HCC-associated pre-S point mutations existed at least 10 years before HCC development, and were more prevalent preceding HCC development in patients from HCC groups than HCC-free group. The number of HCC-associated pre-S point mutations increased over time preceding HCC development, and correlated positively with the time to HCC diagnosis (r = 0.220, p = 0.005).

**Conclusions:**

High prevalence and cumulative evolution of pre-S mutations preceding HCC development suggested a possible carcinogenic role of pre-S mutations and their potential application in HCC risk prediction.

## Introduction

Hepatocellular carcinoma (HCC) is the third major cause of cancer-related mortality in the world, with an estimated annual death rate of 700,000 [1]. Hepatitis B virus (HBV) infection is one of the major causes of HCC. Although the exact role of HBV in hepatocarcinogenesis is not fully understood, several viral factors have been associated with a higher risk of HCC, including viral load, HBV genotype, basal core promoter and precore mutations and pre-S deletions [2, 3].

**Fig 1:**
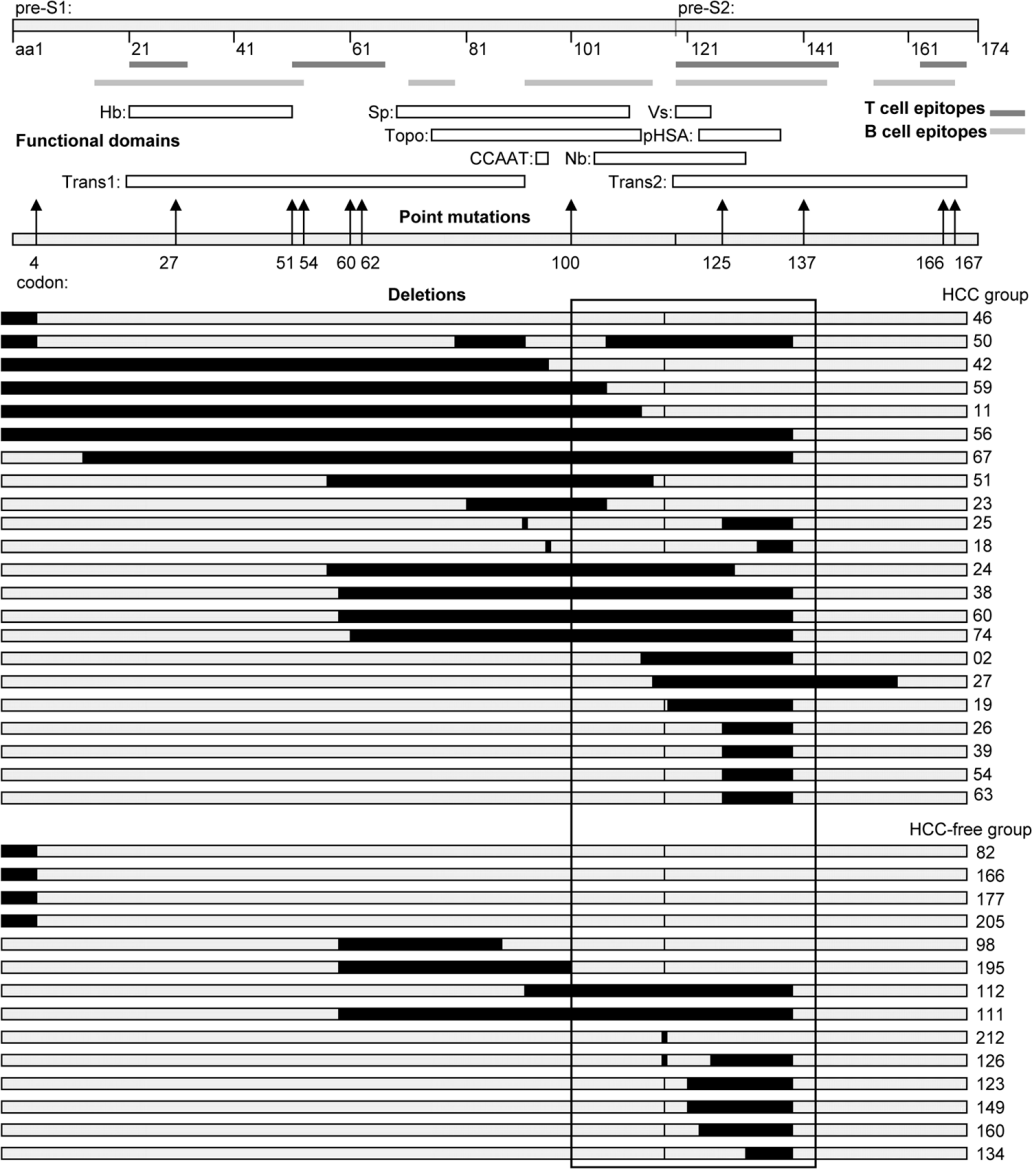
Mapping of pre-S mutations in HCC patients and HCC-free patients. The HBV pre-S region contains immune epitopes and functional domains as depicted. The B-cell epitopes located at aa12-53, aa72-78, aa94-117, aa120-143 and aa157-167 of pre-S gene (overlap regions merged); The T-cell epitopes located at aa21-30, aa52-67, aa120-145 and aa163-172 of pre-S gene (overlap regions merged). The functional domains include the start codons of pre-S1 (aa1) and pre-S2 (aa120), hepatocyte binding site (Hb, aa21-47), S promoter (Sp, nt3045-3180), CCAAT box (nucleotide 3137–3141), topology domain (Topo) includes heat shock protein 70 binding site (aa74-118) and cytosolic anchorage determinant (aa81-105), nucleocapsid binding site (Nb, aa103-127), viral secretion domain (Vs, aa120-124), polymerized human serum albumin binding site (pHSA, aa122-135), transactivator domain (Trans, aa21-90, 120–172). The sequence of each sample is depicted as a block. The black blocks inside represent the deletions of amino acids. The patient identifier numbers are listed at the right.

HBV pre-S region is located at 5’ end of the open reading frames of the surface gene and consists of pre-S1 and pre-S2 domains. Pre-S region contains start codons for the expression of large and middle HBV surface antigens (LHBs and MHBs, respectively), and promoter for the expression of small HBV surface antigen (SHBs). It also contains both B- and T-cell epitopes that mediate immune interactions, hepatocyte binding sites that participate in viral attachment, and other functional domains (Fig 1) [4–9]. Thus, the pre-S region plays multiple and essential roles in HBV replications, infections and viral-host interactions.

Naturally occurring pre-S mutations have been reported during the course of chronic hepatitis B (CHB) infection [10]. Chen et al. [6] compared the prevalence of pre-S deletions in various stages of CHB infection and found that HCC patients have a higher frequency of pre-S deletions. Recent case-control studies have demonstrated that HBV pre-S deletions are independent factors associated with HCC development [11–13]. However, most studies were performed using samples taken after the diagnosis of HCC. The association between pre-S deletions detected prior to development of HCC and risk of HCC remains unclear. In addition, apart from pre-S deletions, point mutations in the pre-S region which are often neglected in most of the studies were also frequently observed in HCC patients [11, 13]. Moreover, there were few studies that longitudinally investigated the evolution of pre-S mutations over the period preceding HCC development.

In the present study, we investigated the association between pre-S mutations and the risk of HCC development using a case-control approach. In addition, we investigated the prevalence and evolutionary changes of HBV pre-S mutations in up to 10 years prior to development of HCC using a longitudinal approach.

## Patients and Methods

### Patients

From July 2007 to December 2012, we identified 116 CHB patients with clinical diagnosis of HCC in our Liver Clinic, Queen Mary Hospital, The University of Hong Kong. Diagnosis of HCC was made by following criteria: (1) positive histology or (2) increasing alpha-fetoprotein (AFP) levels and positive imaging features by computed tomography or magnetic resonance imaging. Of these 116 patients, 74 had stored sera available 1–3 years prior to the time of HCC diagnosis and were enrolled in this study (HCC group). In addition, we enrolled 148 HCC-free CHB patients who were age- and sex-matched to the 74 HCC patients in a 2:1 ratio for case-control comparison (HCC-free group). For the HCC-free patients, all patients have been followed up for at least 3 years after recruitment to exclude the possibility of subsequent HCC development. Serum samples were collected and stored at -20°C until tested.

In addition, 56 of 74 HCC patients and 47 of 148 HCC-free control patients with serum samples collected at earlier time points were longitudinal investigated. We studied the HBV sequence in sera collected at 1–3 years, 4–6 years, 7–9 years and ≥10 years prior to HCC development and at the same time points for the HCC-free group. In the HCC group, a total of 164 serum samples from 56 HCC patients were examined, including 56 (34.2%) samples, 52 (31.7%) samples, 35 (21.3%) and 21 (12.8%) samples at 4 time points of 1–3 years, 4–6 years, 7–9 years and ≥10 years prior to diagnosis of HCC, respectively. In HCC-free group, 136 serum samples from 47 CHB patients were examined, including 47 (34.6) samples, 38 (27.9%) samples, 34 (25%) samples and 17 (12.5%) samples at the same 4 corresponding time points, respectively.

All patients were positive for the hepatitis B surface antigen (HBsAg) for more than 6 months. None was positive for antibody to the hepatitis C virus or had significant alcohol intake. Patients were followed up in our clinic every 6 months for clinical assessment and measurement of alpha-fetoprotein, liver biochemistry, and HBV serology. No written or oral consent was obtained from the participants. The reason was that it was not an interventional study and all data was analyzed anonymously. The study protocol conformed to the ethical guidelines of the 1975 Declaration of Helsinki and was approved by the Institutional Review Board, the University of Hong Kong and West Cluster of Hospital Authority, Hong Kong (Reference Number: UW 14–360).

### HBV DNA extraction, amplification and sequencing

HBV DNA was extracted from 200 μl of serum samples using the PureLink Genomic DNA kit (Life Technologies, Carlsbad, CA) in accordance with manufacturer’s instructions. Semi-nested polymerase chain reaction (PCR) was performed to amplify the entire HBV pre-S region with two sets of nested primers. The outer primers were HBV 2747s (sense: 5’-GTTTACATACTCTGTGGAAGGC-3’ [nucleotide 2747–2767]) and HBV 255a (antisense: 5’-GAGTCTAGACTCTGTGGTATT-3’ [nucleotide 255–235]), and inner primers were HBV 2814s (sense: 5’-GGGTCACCATATTCTTGGGAA-3’ [nucleotide 2814–2834]) and HBV 255a. All PCR reactions were performed with the HotStarTaq Plus DNA Polymerase (Qiagen, Hilden, Germany) in the GeneAmp PCR system 9700 (Applied Biosystems, Foster City, CA) according to the previously described conditions [12]. PCR products were checked by electrophoresis in 1% agarose gel.

The PCR products were purified with the PCRquick-spin PCR Product Purification Kit (iNtRON Biotechnology, Gyeonggi-do, Korea) and then sequenced bi-directionally with the second round primers using the BigDye Terminator v3.1 Cycle Sequencing kit in an ABI PRISM 3500 Genetic Analyzer (Applied Biosystems).

### HBV pre-S sequence analysis

The MEGA 5 software [14] was used to assemble the sense and anti-sense sequences of each PCR product into a contig sequence. All the assembled sequences were aligned with respect to HBV wild-type genotype B and genotype C reference sequences obtained from 54 HBV reference sequences retrieved from the NCBI GenBank (Accession numbers: AB073827-AB073829, DQ995803-DQ995804, DQ089756-DQ0897804). All reference sequences were derived from CHB patients in Hong Kong, with demographic and virologic information available in detail [15–17]. Mutations were identified after comparison with wild-type consensus sequences of genotype B or genotype C. Pre-S deletions were signified by the absence of one or more nucleotides in the aligned sequences [13].

HBV genotypes were determined using NCBI Genotyping tool (http://www.ncbi.nlm.nih.gov/projects/genotyping/formpage.cgi), followed by validation using phylogenetic analysis.

### Detection of Positive selection codons

Datamonkey (http://www.datamonkey.org), a web-server of the HyPhy package, was used to analyze the selection pressures acting on HBV pre-S region [18]. Selection was estimated by comparing the ratio of synonymous and non-synonymous substitutions (dN/dS ratio, or ω) at each codon site in HBV pre-S region. The value of ω <1, ω = 1 and ω >1 indicates negative selection, neutral evolution, and positive selection, respectively. Codons under positive selection were detected via Single likelihood ancestor counting (SLAC) method as implemented in Datamonkey. The positive selection codon was defined when a p value <0.05 was obtained in SLAC method.

### Statistical analysis

Data were presented as mean ± standard deviation or median (range). Univariate analysis of outcome variables was undertaken using the Chi-square test, Fisher’s exact test, the Student’s t-test or spearman correlation as appropriate. Stepwise multiple logistic regression analysis was used to determine the independent factors associated with HCC. Kaplan-Meier analysis was used to calculate the cumulative frequencies of pre-S mutations prior to HCC. All statistical tests were performed using SPSS 16.0 for Windows (SPSS Inc. Chicago, IL, USA). A p-value of <0.05 was considered statistically significant.

## Results

### Demographics and pre-S mutations/ deletion

The demographic and virologic characteristics of patients in HCC group and HCC-free group are depicted in Table 1. There were no statistically significant differences in age, gender and HBeAg status between the two groups. The majority of patients were older than 50 years (81.1%), male (79.7%) and hepatitis B e antigen (HBeAg)-negative (82.4%). All patients harbored either HBV genotype B or C. Genotype C was found at a higher prevalence in the HCC group than in the HCC-free group (89.2% vs. 50.7%, p<0.001). Thirty-six of 74 (48.6%) HCC patients and 3 of 148 (2.0%) HCC-free patients had received anti-viral therapy. Fifty-six of 74 (75.7%) HCC patients and 32 of 148 (21.6%) HCC-free patients had liver cirrhosis. The HCC patients had higher ALT and total bilirubin levels than the HCC-free patients (p = 0.009 and p = 0.021, respectively).

**Table 1:** Comparison of demographic and virologic characteristics for HCC patients and control HCC-free patients.

Variable	HCC (%)	HCC-free (%)	P
N	74	148	
Age(years)	58.9±10.0	58.8±10.5	NS
<50	14 (18.9)	28 (18.9)	
>50	60 (80.1)	120 (80.1)	
Gender			NS
Female	15 (20.3)	30 (20.3)	
Male	59 (79.7)	118 (79.7)	
HBeAg status			NS
Positive	15 (20.3)	24 (16.2)	
Negative	59 (79.7)	124 (83.8)	
HBV genotype			<0.001
B	8 (10.8)	73 (49.3)	
C	66 (89.2)	75 (50.7)	
Antiviral treated	36 (48.6)	3 (2.0)	<0.001
Cirrhosis	56 (75.7)	32 (21.6)	<0.001
ALT (U/L)	60.7±60.9	40.9±36.4	0.009
Total Bilirubin (μmol/L)	14.3±9.9	12.6±6.0	0.021
Pre-S deletions	22 (29.7)	14 (9.5)	<0.001
W4m	11 (14.9)	8 (5.4)	0.018
DG27m	56 (75.7)	41 (27.7)	<0.001
NQ51m	46 (62.2)	27 (18.2)	<0.001
DA54m	45 (60.8)	37 (25)	<0.001
V60m	39 (52.7)	21 (14.2)	<0.001
AS62m	41 (55.4)	23 (15.5)	<0.001
Q100m	32 (43.2)	11 (7.4)	<0.001
TS125m	42 (56.8)	32 (21.6)	<0.001
R137m	30 (40.5)	11 (7.4)	<0.001
S166m	34 (45.9)	12 (8.1)	<0.001
KR167m	36 (48.6)	22 (14.9)	<0.001

NOTE. Variables were at the sampling time for HCC-free patients or the nearest sampling time to diagnosis of HCC for HCC patients. Data are no. (%) of subjects or mean ± standard deviation. HCC, hepatocellular carcinoma; NS, not significant; ALT, Alanine Aminotransferase. To interpret the mutations listed, take DG27m for example, D indicates the wild type amino acid in codon 27 of genotype B while G indicates the wild type amino acid in codon 27 of genotype C, and m indicates amino acid substitution mutations.

HBV pre-S deletions and point mutations that displayed a preferential distribution between HCC and HCC-free groups are listed in Table 1. Pre-S deletions were detected in a higher proportion in HCC patients (22/74, 29.7%) than in HCC-free patients (14/148, 9.5%) (p<0.001). Higher frequencies of pre-S point mutations at 11 codons (codons 4, 27, 51, 54, 60, 62, 100, 125, 137, 166 and 167) were also observed in the HCC patients (range: 14.9–75.7%) than in the HCC-free patients (range: 5.4–27.7%; p<0.05 for all comparisons). To explore the association between pre-S mutations and cirrhosis, we compared the frequencies of pre-S mutations between the 32 HCC-free patients with cirrhosis and the 116 patients without cirrhosis. There was no significant difference in the frequencies of pre-S deletions, as well as point mutations at the 11 codons, between two groups (all p>0.05, data not shown). We also found that the frequencies of pre-S deletions and point mutations at the 11 codons between the treated and non-treated patients were comparable (all p>0.05, data not shown).

### Mapping of HCC-associated pre-S mutations

The location and size of pre-S deletions in the 14 HCC-free patients and 22 HCC patients are depicted in Fig 1. All the pre-S deletions were in-frame deletions, with deletions ranging from 3 to 422 base pairs. Most of the pre-S deletions encompassed B- and T-cell epitopes and important functional domains, such as pre-S1 start codon, pre-S2 start codon, hepatocyte binding site, S promoter, CCAAT box, topology domain, nucleocapsid binding site and polymerized human serum albumin binding site. Interestingly, more than half (21/36; 58.3%) of the samples had pre-S deletions ended at aa140–142 of the pre-S region. Moreover, the majority of samples (30/36; 83.3%) had pre-S deletions which encompassed the pre-S region between amino acid (aa) 100 and aa 140, suggesting that this region may be a hot-spot region for deletions occurrence. In particular, of the 22 HCC patients with pre-S deletions, all but 2 patients had pre-S deletions encompassing part or all of these regions.

The positions of the 11 pre-S point mutations that were more frequently found in HCC patients are illustrated in Fig 1. Immune epitope mapping of these mutations showed that point mutations located in B-cell epitope (mutations at codon 51 and 100), or T-cell epitope (mutations at codon 54, 60 and 62), or both B- and T-cell epitopes (mutations at codon 27, 125, 137, 166 and 167). Of these 11 point mutations, only mutation at codon 4 was found outside the B- and T-cell epitopes. Further functional mapping of these mutations showed that amino acid changes might impact functional domains of hepatocyte binding site, S promoter, heat shock protein 70 binding site, cytosolic anchorage determinant, nucleocapsid binding site, polymerized human serum albumin binding site and transactivator.

### Independent risk factors

We performed regression analysis to determine the independent effects of the factors of HBeAg status, HBV genotype, cirrhosis status, the presence or absence of pre-S deletions, the 11 pre-S point mutations, and ALT and total bilirubin levels on the risk of HCC development. Multiple logistic regression analysis showed that pre-S deletions (OR, 8.253; 95% CI, 2.668–25.531; p<0.001) and 2 pre-S point mutations at codon 51 (OR, 13.917; 95% CI, 4.957–39.075; p<0.001), codon 167 (OR, 7.880; 95% CI, 2.717–22.852; p<0.001), ALT level (OR, 1.014; 95% CI, 1.002–1.025; p = 0.018) and cirrhosis (OR, 0.058; 95% CI, 0.022–0.157; p<0.001) were independent risk factors to the development of HCC (Table 2). HBeAg status, HBV genotype, total bilirubin level, and the rest of the 9 pre-S point mutations were not independent factors associated with HCC development (all p>0.05).

**Table 2:** Independent risk factors associated with HCC development.

	OR	95% CI	P value
Pre-S deletions	8.253	2.668–25.531	<0.001
NQ51m	13.917	4.957–39.075	<0.001
KR167m	7.880	2.717–22.852	<0.001
ALT	1.014	1.002–1.025	0.018
Cirrhosis	0.058	0.022–0.157	<0.001

Note. OR: odds ratios of HCC-related factors; 95% CI, 95% confidence intervals.

### Positive selection codons detection

**Fig 2:**
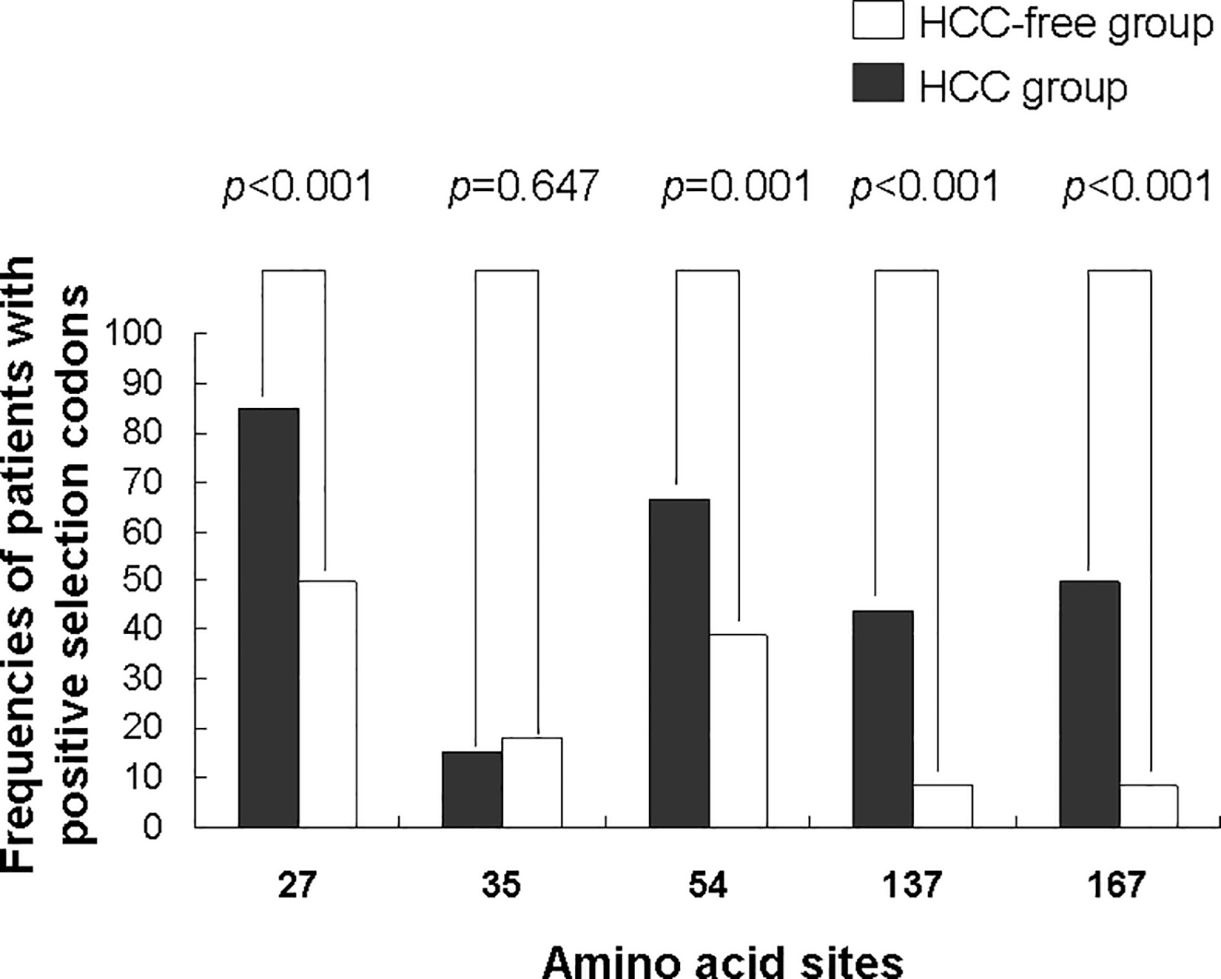
The positive selection codons detected in genotype C sequences. Prevalence of mutations at positive selected sites of genotype C sequences in HCC and HCC-free group.

Positive selection codons that computed by SLAC method are depicted in Fig 2. We identified 5 amino acid sites (codon 27, 35, 54, 137 and 167) which were under positive selection pressure in genotype C sequences, while no positive selection codon was detected in genotype B sequences. Immune epitope mapping of these mutations suggested that all of these amino acid substitutions were located in B- or/and T-cell epitopes. In particular, frequencies of amino acid substitutions at codon 27, 54, 137 and 167 were significantly higher in HCC group than HCC-free group (p<0.05 for all comparisons).

### Pre-S mutations longitudinal study

**Fig 3:**
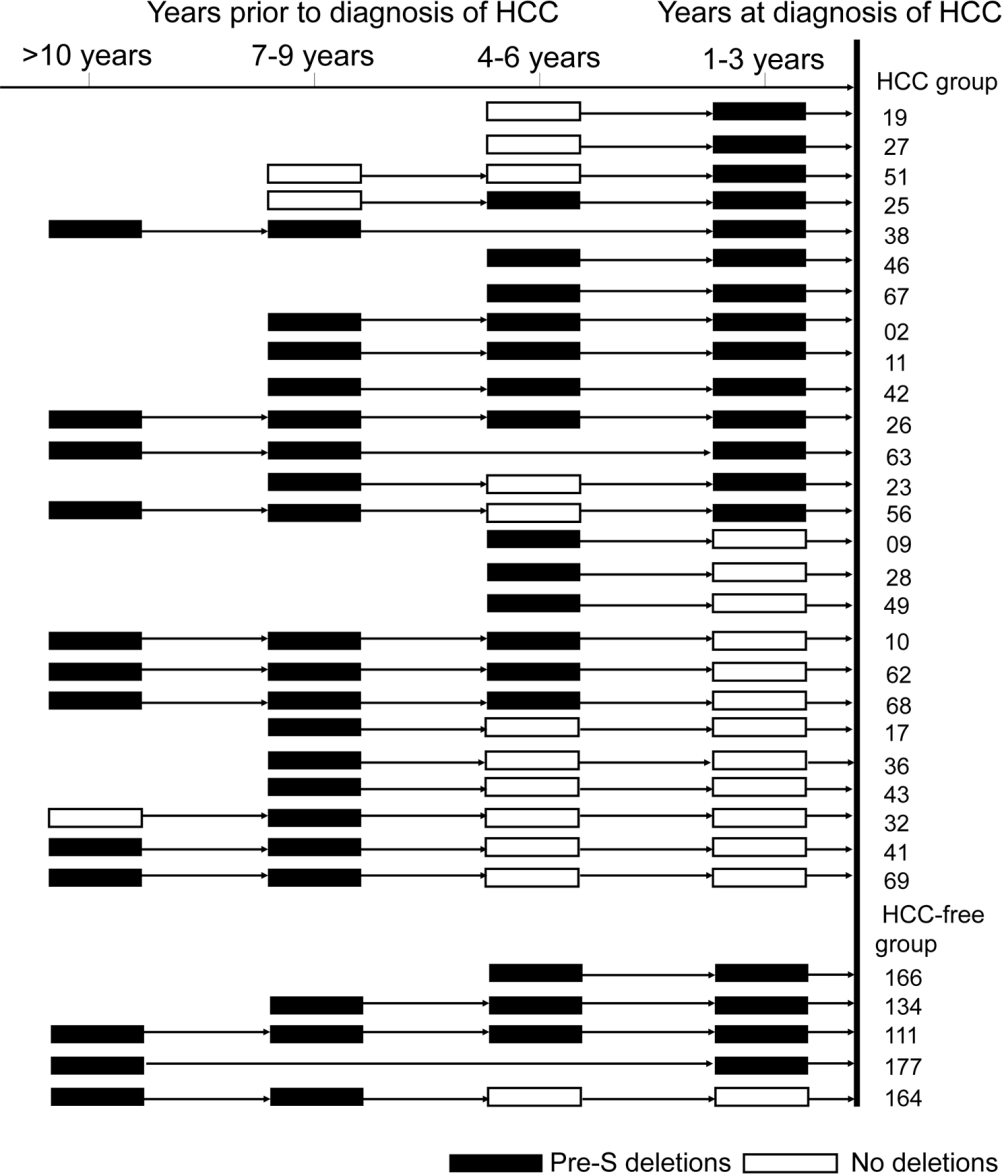
Longitudinal observation of pre-S deletions over time prior to HCC development. Each sequence is depicted as a block and its location relative to the timeline indicates the time points prior to diagnosis of HCC. Empty blocks represent sequences without pre-S deletions; whereas black blocks represent sequences with pre-S deletions. The patient identifier numbers are listed at the right.

Fifty-six HCC patients and 47 HCC-free patients had serial serum samples available for longitudinal analysis. The changes in frequencies of pre-S deletions and the 11 pre-S point mutations over time in these 103 patients were depicted in Table 3. In the 56 HCC patients, pre-S deletions were detected in 25%, 25%, 48.6%, and 42.9% of HCC patients at 1–3 years, 4–6 years, 7–9 years, and ≥10 years prior to HCC development, respectively. In the HCC-free group, the frequencies of pre-S deletions at the 4 corresponding time points were lower (8.5%, 7.9%, 8.8%, and 17.6%, respectively; with p values of 0.037, 0.05, <0.001, and 0.161, respectively). As depicted in Fig 3, of the 14 HCC patients detected with pre-S deletions at 1–3 years before HCC, pre-S deletions were also detected in 7 patients at 4–6 years earlier, in 8 patients at 7–9 years earlier, and in 4 patients at ≥10 years before HCC. However, in 12 HCC patients, pre-S deletions were detected only at earlier time points (4–6 years, 7–9 years, or ≥10 years), but not detected at 1–3 years prior to HCC. Taken together, a significantly higher cumulative frequencies of pre-S deletion were observed in the HCC group than HCC-free group (16.1% vs. 6.4% at ≥10 years, 30.4% vs. 8.5% at 7–9 years, 41.1% vs. 10.6% at 4–6 years, and 46.4% vs. 10.6% at 1–3 years prior to HCC, respectively, p<0.001).

**Table 3:** Pre-S deletions/point mutations at time points assessed prior to HCC development.

Years prior to HCC		≥10 years	7–9 years	4–6 years	1–3 years
Pre-S deletions	HCC	9/21 (42.9%)	17/35 (48.6%)	13/52 (25%)	14/56 (25%)
	HCC-free	3/17 (17.6%)	3/34 (8.8%)	3/38 (7.9%)	4/47 (8.5%)
	P value	0.161	<0.001	0.05	0.037
W4m	HCC	0/21 (0%)	0/35 (0%)	5/52 (9.6%)	6/56 (10.7%)
	HCC-free	0/17 (0%)	2/34 (5.9%)	5/38 (13.2%)	4/47 (8.5%)
	P value	1	0.239	0.737	0.752
DG27m	HCC	12/21 (57.1%)	22/35 (62.9%)	36/52 (69.2%)	43/56 (76.8%)
	HCC-free	6/17 (35.3%)	7/34 (20.6%)	7/38 (18.4%)	11/47 (23.4%)
	P value	0.180	<0.001	<0.001	<0.001
NQ51m	HCC	9/21 (42.9%)	17/35 (48.6%)	35/52 (67.3%)	35/56 (62.5%)
	HCC-free	5/17 (29.4%)	8/34 (23.5%)	11/38 (28.9%)	9/47 (19.1%)
	P value	0.393	0.030	<0.001	<0.001
DA54m	HCC	10/21 (47.6%)	15/35 (42.9%)	32/52 (61.5%)	36/56 (64.3%)
	HCC-free	3/17 (17.6%)	6/34 (17.6%)	10/38 (26.3%)	14/47 (29.8%)
	P value	0.086	0.023	0.001	<0.001
V60m	HCC	9/21 (42.9%)	14/35 (40%)	34/52 (65.4%)	34/56 (60.7%)
	HCC-free	3/17 (17.6%)	4/34 (11.8%)	8/38 (21.1%)	8/47(17.0%)
	P value	0.161	0.013	<0.001	<0.001
AS62m	HCC	8/21 (38.1%)	15/35 (42.9%)	30/52 (57.7%)	34/56 (60.7%)
	HCC-free	3/17 (17.6%)	5/34 (14.7%)	8/38 (21.1%))	9/47 (19.1%)
	P value	0.282	0.010	0.001	<0.001
Q100m	HCC	7/21 (33.3%)	11/35 (31.4%)	27/52 (51.9%)	30/56 (53.6%)
	HCC-free	0/17 (0%)	1/34 (2.9%)	3/38 (7.9%)	4/47 (8.5%)
	P value	0.011	0.003	0.001	<0.001
TS125m	HCC	10/21 (47.6%)	17/35 (48.6%)	35/52 (67.3%)	37/56 (66.1%)
	HCC-free	4/17 (23.5%)	7/34 (20.6%)	12/38 (31.6%)	13/47 (27.7%)
	P value	0.181	0.015	0.001	<0.001
R137m	HCC	6/21 (28.6%)	10/35(28.6%)	28/52 (53.8%)	27/56 (48.2%)
	HCC-free	0/17 (0%)	1/34 (2.9%)	3/38 (7.9%)	3/47 (6.4%)
	P value	0.024	0.006	<0.001	<0.001
S166m	HCC	6/21 (28.6%)	13/35 (37.1%)	29/52 (55.8%)	32/56 (57.1%)
	HCC-free	1/17 (5.9%)	3/34 (8.8%)	5/38 (13.2%)	6/47 (12.8%)
	P value	0.104	0.009	<0.001	<0.001
KR167m	HCC	5/21 (23.8%)	9/35 (25.7%)	26/52 (50%)	32/56 (57.1%)
	HCC-free	2/17 (11.8%)	5/34 (14.7%)	7/38 (18.4%)	9/47 (19.1%)
	P value	0.427	0.256	0.002	<0.001

Note. To interpret the mutations listed, take DG27m for example, D indicates the wild type amino acid in codon 27 of genotype B while G indicates the wild type amino acid in codon 27 of genotype C, and m indicates amino acid substitution mutations. Data are no./no.(%) of subjects detected with mutations/ total subjects unless otherwise specified.

Next, we focused on the 11 pre-S point mutations that were identified more frequently in HCC patients in our case-control study. Of these 11 pre-S mutations, 10 (except codon 4) were also found at higher frequencies in the HCC group than the HCC-free group at 1–3 years prior to HCC development in our longitudinal cohort. All these 10 pre-S mutations existed ≥10 years before HCC. Among these 10 mutations, the number of mutated codons with differential distribution between the HCC and HCC-free patients increased with time prior to HCC: a significantly higher frequency of mutations was detected in the HCC patients in 2/10 codons at ≥10 years, in 9/10 codons at 7–9 years, in 10/10 codons at 4–6 years, and 10/10 codons at 1–3 years prior to HCC.

**Fig 4:**
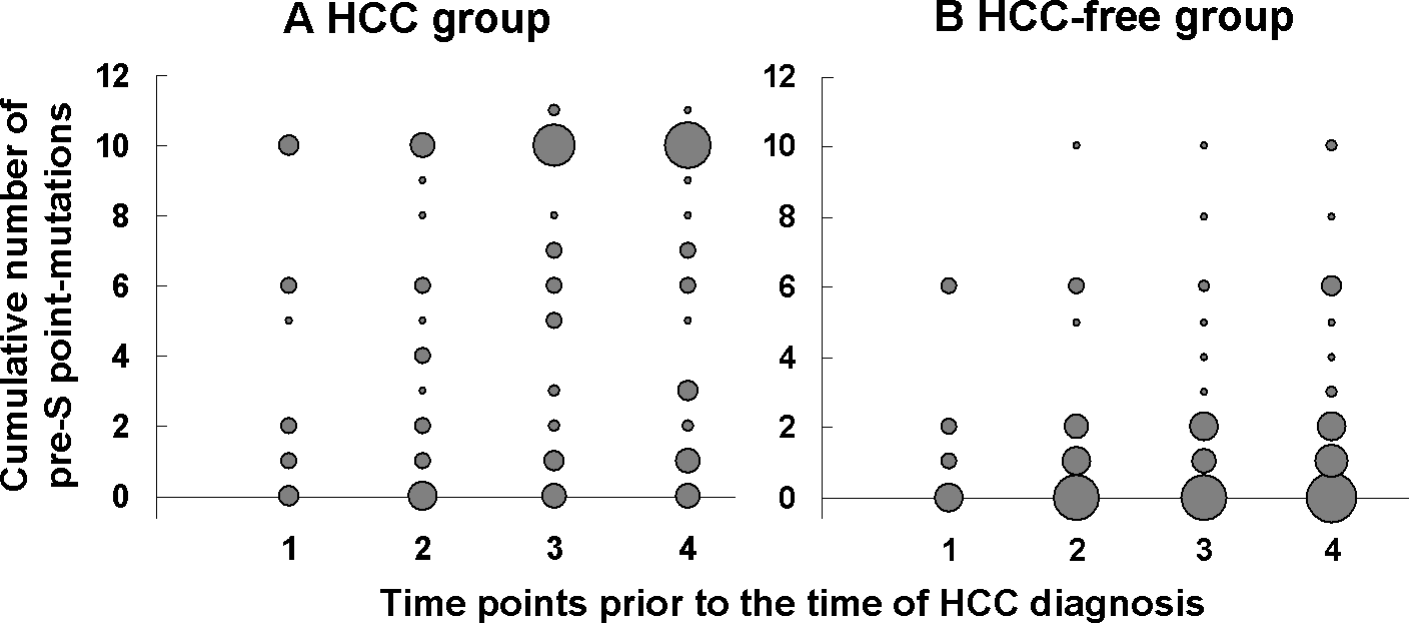
Changes of HBV pre-S point mutations numbers prior to HCC development. Bubble plot of cumulative number of HBV pre-S point mutations associated with HCC at 4 time points in HCC group (A) and HCC-free group (B). Time points 1–4 represent time points of more than ≥10 years, 7–9 years, 4–6 years and 1–3 years before HCC diagnosis, respectively. The size of the circles corresponds to the number of patients at each given time point detected with each given number of point mutation.

The accumulation of HCC-associated pre-S point mutations were illustrated in Fig 4A. The number of pre-S point mutations in the HCC patients increased over time preceding HCC development, and correlated positively with the time to HCC diagnosis (spearman correlation coefficient = 0.220; p = 0.005). On the other hand, the bubble plot of HCC-free group showed the prevalence of pre-S point mutations was relatively low at 4 matched time points (Fig 4B). Although the number of pre-S point mutations tended to increase over time, there was no correlation between the time and number of mutations in the HCC-free group (spearman correlation coefficient = 0.073; p = 0.398).

## Discussion

The present study is the first to investigate the prevalence and changes of pre-S mutations with a long term (as long as 10 years) follow-up prior to HCC development. We identified pre-S deletions and 11 pre-S point mutations that were more frequently detected in HCC patients with a case-control approach. Using a longitudinal approach, we further showed that these pre-S deletions/mutations already existed at least 10 years before the diagnosis of HCC and were highly prevalent prior to HCC diagnosis. Prior to HCC development, an increase occurrence of pre-S point mutations over time was also observed whereas this phenomenon was not observed in the controls without development of HCC.

Our finding that pre-S deletion was an independent risk factor for HCC development was consistent with most previous studies [6, 11, 12]. The hepatocarcinogenic role of pre-S deletions has been suggested by previous studies. Both pre-S1 and pre-S2 deletions can cause accumulation of LHBs proteins and viral particles in the endoplasmic reticulum (ER), and subsequently induce ER stress and oxidative DNA damage of HBV-infected hepatocytes, which may involved in the hepatocarcinogenesis [19]. In addition, LHBs and truncated middle surface protein have been recognized to be transcriptional activators which may initiate the Ras/Raf-1/ERK signaling, and hence be another oncogenic mechanism [20]. A recent study also demonstrates an enhanced expression of VEGF-A and activation of Akt/mTOR signaling in ground glass hepatocytes harboring pre-S deletion mutants, thus providing another potential mechanism for tumorigenesis [21].

Unlike pre-S deletions, pre-S point mutations in relation to HCC have been not well characterized. In the present study, we identified 11 pre-S point mutations that were highly associated with HCC development. Consistent with our study, Chen et al. [11] also found that point mutation at codon 4 in pre-S region is associated with HCC by a case-control study. In addition, Yin et al. [13] also demonstrated higher frequencies of point mutations at codon 4, 60 and 125 in the pre-S region in HCC patients. The impact of various HBV pre-S point mutations on HCC development remains unclear. As observed in Fig 1, functional mapping of pre-S mutations suggested that those point mutations frequently occurred in the B- and T-cell epitopes and functional domains. Point mutations in the S promoter and the CCAAT binding factors could lead to decreased synthesis of surface protein, resulting in intracellular retention of envelope proteins and ER stress, thus possibly inducing HCC carcinogenesis [22–24]. To fully explore the relationship between HBV pre-S mutations and HCC development, further elucidation of molecular mechanism focusing on hepatocyte transformation through construction of liver cell lines expressing these pre-S mutations are needed.

In our study, of the 5 codons under positive selection, the frequencies of point mutations at codon 27, 54, 137 and 167 were significantly higher in the HCC group than HCC-free group. All these codons were within B- and T- cell epitopes, while alternations in these immune target sites could lead to escape from immune surveillance and result in persistent infection [24]. It is likely that these point mutations were positively selected by immune pressure during HCC development. This is supported by our longitudinal results which showed accumulation of these mutations over time before HCC development. Thus, our results suggested that the positive selection might be responsible for the generation of HCC-associated point mutations.

To our best knowledge, up to now, there are only limited longitudinal studies of pre-S mutations in relation to the development of HCC. Qu et al. [25] showed that complex HBV mutations (pre-S deletions, T1762/A1764, and T1766 and/or A1768 mutants) were gradually combined during the development of HCC. Our previous study [12] also reported the emergence of de novo pre-S deletions before the development of HCC. However, the 2 above-mentioned studies were limited by the small number of HCC patients and the short follow-up time. In the current study, we recruited a large number of patients with longer follow-up duration and found that a high prevalence of pre-S deletions and point mutations was detected at up to 10 years prior to HCC development. Although the frequency of pre-S deletions seemed to progressively decrease prior to HCC, this might be due to the markedly increasing number of available serum samples from 10 years before HCC to the time near HCC diagnosis. To verify this, we performed a Kaplan-Meier analysis and found that there was a significantly increased cumulative frequency of pre-S deletions prior to HCC, which strongly supported the carcinogenetic role of pre-S deletions. In addition, we identified that there was an increasing trend of the number of pre-S point mutations over time before the development of HCC. Taken together, our results suggest that, in addition to pre-S deletion, these pre-S mutations might also be involved in the pathogenesis of HCC. In addition, early detection of pre-S deletions and point mutations as early as 10 years prior to HCC may potentially permit the formulation of a predictive HCC risk score for CHB patients.

The present study has several limitations. First, it is not possible to determine the exact time of the emergence of HCC. In the current study, we used the time of clinical diagnosis of HCC. Depending on the frequency of follow-up, the exact time of the emergence of HCC may differ. Nevertheless, like all other clinical studies, this may be the best possible estimation. Secondly, data for HBV DNA, a known risk factor for HCC prediction, were not available. Finally, we determined pre-S mutations via direct sequencing method, which could not detect mutations that existed as minority below 20% of the viral quasi-species [26]. In this study, the discontinuous observation of pre-S deletions during HCC development might be a result of HBV variants harboring pre-S deletions evolving as minority of quasispecies and were missed by direct sequencing. Thus, the prevalence of pre-S mutations might be underestimated, and further studies using more sensitive sequencing techniques are encouraged.

In conclusion, our current study identified a high prevalence of pre-S deletions and point mutations prior to HCC development. In particular, pre-S deletions, and point mutations at pre-S codons 51 and 167 were independent factors associated with HCC development. Pre-S mutations existed at least 10 years before the diagnosis of HCC, and the number of mutations increased over time. Our findings suggested the carcinogenic role of pre-S mutations and their potential application in HCC risk prediction. The effect of individual pre-S mutations on HCC development warrants further studies.
